# Secretome proteomics reveals candidate non-invasive biomarkers of *BRCA1* deficiency in breast cancer

**DOI:** 10.18632/oncotarget.11535

**Published:** 2016-08-23

**Authors:** Marc Warmoes, Siu W. Lam, Petra van der Groep, Janneke E. Jaspers, Yvonne H.C.M. Smolders, Leon de Boer, Thang V. Pham, Sander R. Piersma, Sven Rottenberg, Epie Boven, Jos Jonkers, Paul J. van Diest, Connie R. Jimenez

**Affiliations:** ^1^ Oncoproteomics Laboratory, Department of Medical Oncology, VU University Medical Center, Amsterdam, The Netherlands; ^2^ Department of Pathology, University Medical Center Utrecht, Utrecht, The Netherlands; ^3^ Department of Internal Medicine, University Medical Center Utrecht, Utrecht, The Netherlands; ^4^ Division of Molecular Oncology, The Netherlands Cancer Institute, Amsterdam, The Netherlands; ^5^ Division of Molecular Pathology, The Netherlands Cancer Institute, Amsterdam, The Netherlands; ^6^ Institute of Animal Pathology, Vetsuisse Faculty, University of Bern, Bern, Switzerland

**Keywords:** proteomics, biomarkers, BRCA1, breast cancer

## Abstract

Breast cancer arising in female *BRCA1* mutation carriers is characterized by an aggressive phenotype and early age of onset. We performed tandem mass spectrometry-based proteomics of secretomes and exosome-like extracellular vesicles from *BRCA1*-deficient and *BRCA1*-proficient murine breast tumor models to identify extracellular protein biomarkers, which can be used as an adjunct to current diagnostic modalities in patients with *BRCA1*-deficient breast cancer. We identified 2,107 proteins, of which 215 were highly enriched in the *BRCA1*-deficient secretome. We demonstrated that *BRCA1*-deficient secretome proteins could cluster most human *BRCA1*- and BRCA2-related breast carcinomas at the transcriptome level. Topoisomerase I (TOP1) and P-cadherin (CDH3) expression was investigated by immunohistochemistry on tissue microarrays of a large panel of 253 human breast carcinomas with and without *BRCA1/2* mutations. We showed that expression of TOP1 and CDH3 was significantly increased in human *BRCA1*-related breast carcinomas relative to sporadic cases (*p* = 0.002 and *p* < 0.001, respectively). Multiple logistic regression showed that TOP1 (adjusted odds ratio [OR] 3.75; 95% confidence interval [95% CI], 1.85 - 7.71, *p* < 0.001) as well as CDH3 positivity (adjusted OR 2.45; 95% CI, 1.08 - 5.49, *p* = 0.032) were associated with *BRCA1/2*-related breast carcinomas after adjustment for triple-negative phenotype and age. In conclusion, proteome profiling of secretome using murine breast tumor models is a powerful strategy to identify non-invasive candidate biomarkers of *BRCA1*-deficient breast cancer. We demonstrate that TOP1 and CDH3 are closely associated to *BRCA1*-deficient breast cancer. These data merit further investigation for early detection of tumors arising in *BRCA1* mutation carriers.

## INTRODUCTION

Approximately 5–10% of female breast cancer are believed to be hereditary, caused by a germline mutation in the *BRCA1* or *BRCA2* genes. Owing to an increased lifetime cancer risk and young age of onset, mutation carriers are eligible for an intensified surveillance program consisting of clinical breast examination and annual screening mammography with the intention to detect breast cancer at a potentially curable stage. The accuracy of screening mammography to detect early breast lesions is, however, limited in young women due to their dense breast tissue [1–3]. Magnetic resonance imaging as an adjunct to screening mammography has improved sensitivity to detect suspected breast lesions [4, 5], but interval breast cancer rates remain substantial and specificity is limited [6]. There is therefore a compelling need to improve current clinical management i.e. early breast cancer detection in *BRCA* mutation carriers.

Tumor cell-secreted proteins, constituting the secretome, have been proposed as biomarkers potentially detectable in the blood circulation or other body fluids [7]. Proteomic profiling of cancer secretomes from genetically engineered mouse models (GEMMs) with tissue-specific deletion of targeted genes may point towards relevant proteins, that are closely associated with the altered gene. For example, employing proteomic analysis on conditional GEMMs for *BRCA1*-deficient and -proficient breast tumors, we have previously identified a tissue-specific signature of 45 proteins, that can discriminate human *BRCA1*- and *BRCA2*-deficient breast carcinomas from other familial or sporadic breast carcinomas [8–10]. The question remains whether this signature is applicable for routine clinical assessment, but our findings, together with other reports, underscore the usefulness of GEMMs and the potential of proteomics as an approach for discovery of novel protein biomarkers.

In the current study, we applied an in-depth proteomic profiling of secretomes and extracellular vesicles (EVs) of *BRCA1* -deficient and *-*proficient breast tumor cell lines derived from three validated GEMMs. Comparative analysis revealed that *BRCA1* deficiency has a marked effect on the repertoire of released proteins. Moreover, the majority of these proteins has been shown to be detectable in human plasma, indicative of their potential as blood-based markers. As a first step to evaluate clinical relevance, we analyzed protein expression of topoisomerase I (TOP1) and P-cadherin (CDH3) in a large panel of breast cancer tissues from *BRCA1/2* mutation carriers and women without a hereditary predisposition. We report that TOP1 and CDH3 were expressed to a higher extent in *BRCA1*-related breast carcinomas relative to sporadic breast carcinomas, highlighting their potential clinical usefulness for breast cancer detection in women with a *BRCA1* mutation.

## RESULTS

### Protein profiling of *BRCA1*-deficient and -proficient breast tumor cell line secretomes

**Figure 1:**
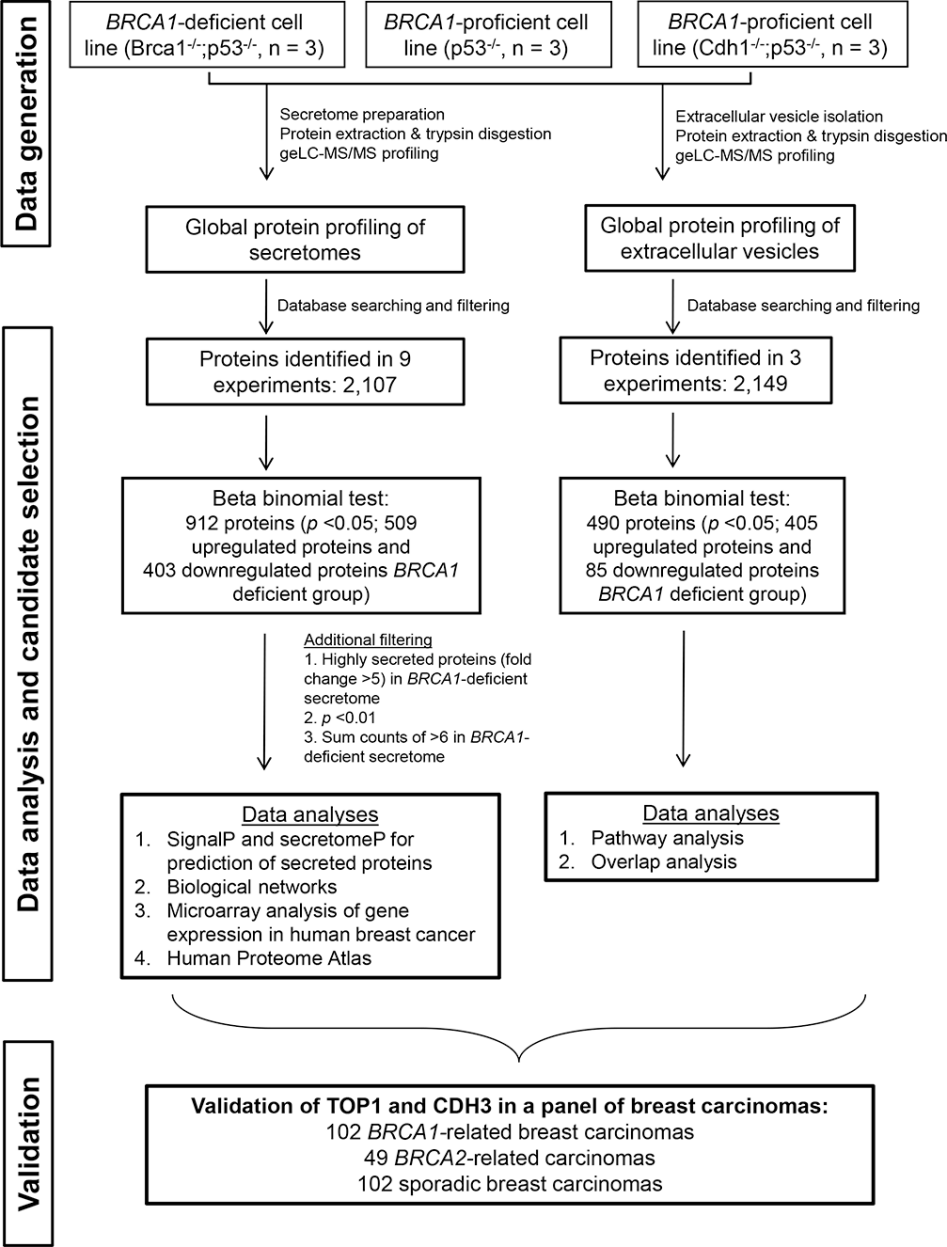
Experimental workflow to identify and validate *BRCA1* deficiency protein biomarkers The discovery experiment included three groups consisting of one *BRCA1*-deficient model and two *BRCA1*-proficient models, with three animals in each group.

For this study, we used primary mammary tumor cell lines derived from a well-characterized GEMM for *BRCA1*-deficient breast cancer (K14*Cre*;*Brca*^−/−^;*p53*^−/−^) and from two different GEMMs for *BRCA1*-proficient breast cancer (K14*Cre*;*p53*^−/−^ and K14*Cre*;*Cdh1*^−/−^;*p53*^−/−^). To identify secreted proteins potentially associated with *BRCA1* deficiency, we analyzed secretomes (in biological triplicate for each cell line) using a robust and reproducible label-free proteomics workflow [8, 11] as shown in Figure 1. Reproducibility of protein identification and quantification from biological triplicate experiments was high (an overlap of identified proteins of > 80% and the coefficient of variation of 14–15% of the overlapping proteins across triplicate experiments of each group, Supplementary Figure 1A).

A total of 2,107 proteins were identified in at least one of nine secretome samples. A large proportion of proteins (59%) was predicted to be secreted via classical (24–32%) or non-classical (29–33%) secretory pathways as determined with SignalP and SecretomeP (Supplementary Figure 2 and Supplementary Table 1). Of 2,107 proteins, 1,365 proteins were commonly identified among the three cell lines (in at least one triplicate, see Supplementary Figure 1B). Unsupervised hierarchical clustering analysis on all proteins demonstrated a distinct separation between *BRCA1*-deficient and *BRCA1*-profient groups indicating that the proteomic portrait of secretome may be influenced by the *BRCA1* gene knockout status (Supplementary Figure 3).

A total of 912 significantly differentially released proteins between *BRCA1*-deficient and *BRCA1*-proficient secretomes were identified. Of these, 509 proteins were more abundantly present in *BRCA1*-deficient secretomes, whereas 403 proteins were less abundantly released (Supplementary Table 1).

### Murine *BRCA1*-deficient proteins in human *BRCA1/2*-mutated breast cancer

**Figure 2:**
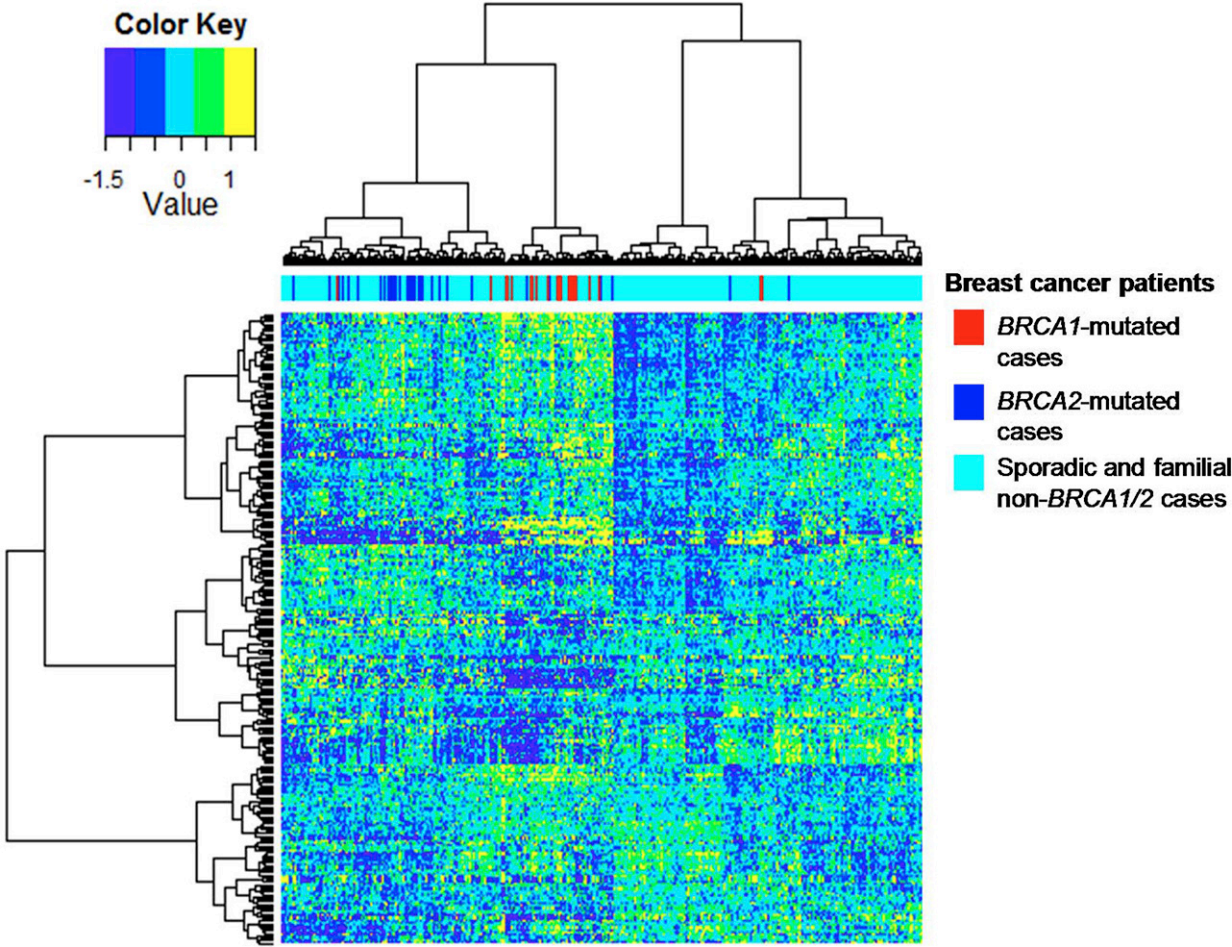
Hierarchical cluster analysis of the mRNA dataset for *BRCA1/2* breast carcinomas on the basis of gene expression of mapped *BRCA1*-deficient proteins Hierarchical clustering of 254 upregulated *BRCA1*-deficient proteins showed a separation of most human *BRCA1/2*-mutated breast carcinomas from sporadic breast carcinomas, when mapped to the mRNA dataset as published by Jönsson et al (Breast Cancer Res 2010).

To examine human relevance, we analyzed the 509 upregulated *BRCA1*-deficient proteins in an mRNA dataset as published by Jönsson et al. [12]. This dataset contains 359 patients with breast cancer, among which there were 22 *BRCA1* mutation cases, 32 *BRCA2* mutation cases and 305 sporadic cases. Among the upregulated *BRCA1*-deficient proteins, 254 proteins could be mapped to the corresponding mRNA transcript levels. Hierarchical cluster analysis showed that the mapped mRNA transcripts could separate the majority of the *BRCA1/2* -deficient breast carcinomas from the majority of sporadic breast carcinomas (Figure 2). A total of 50 mRNA transcripts were also overexpressed in *BRCA1/2*-deficient breast cancer relative to sporadic breast cancer (*p* < 0.1, Supplementary Table 2). These included chromatin remodeling proteins including SMC2, SMC1A, TOP2A, DNMT1 and DEK. Two candidates, namely SMC1A and TOP2A, were part of our previously identified 45 proteins BRCA-like tissue signature [8]. Although protein abundance and mRNA expression may not consistently correlate because of e.g. post transcriptional regulation, our findings suggest that proteins highly released by *BRCA1*-deficient breast cancer cell line, when mapped to mRNA transcript, may enrich *BRCA1/2*-related breast cancer.

### Selection of candidate non-invasive *BRCA1*-deficiency breast tumor biomarkers and in silico exploration of their connectivity and biological functions

To reduce the list of differentially released proteins to the most discriminatory ones, we applied more stringent selection criteria (*p* < 0.01, fold change > 5 and total spectral count of ≥ 6 in biological triplicates), resulting in 215 proteins highly enriched in the *BRCA1*-deficient secretome and 100 proteins highly enriched in the *BRCA1*-proficient secretome (Supplementary Table 2). Interestingly, the proportion of nuclear proteins was higher in these 215 *BRCA1*-deficient proteins compared to that in the 100 *BRCA1*-proficient proteins (25 vs 14%, Supplementary Figure 4).

**Figure 3:**
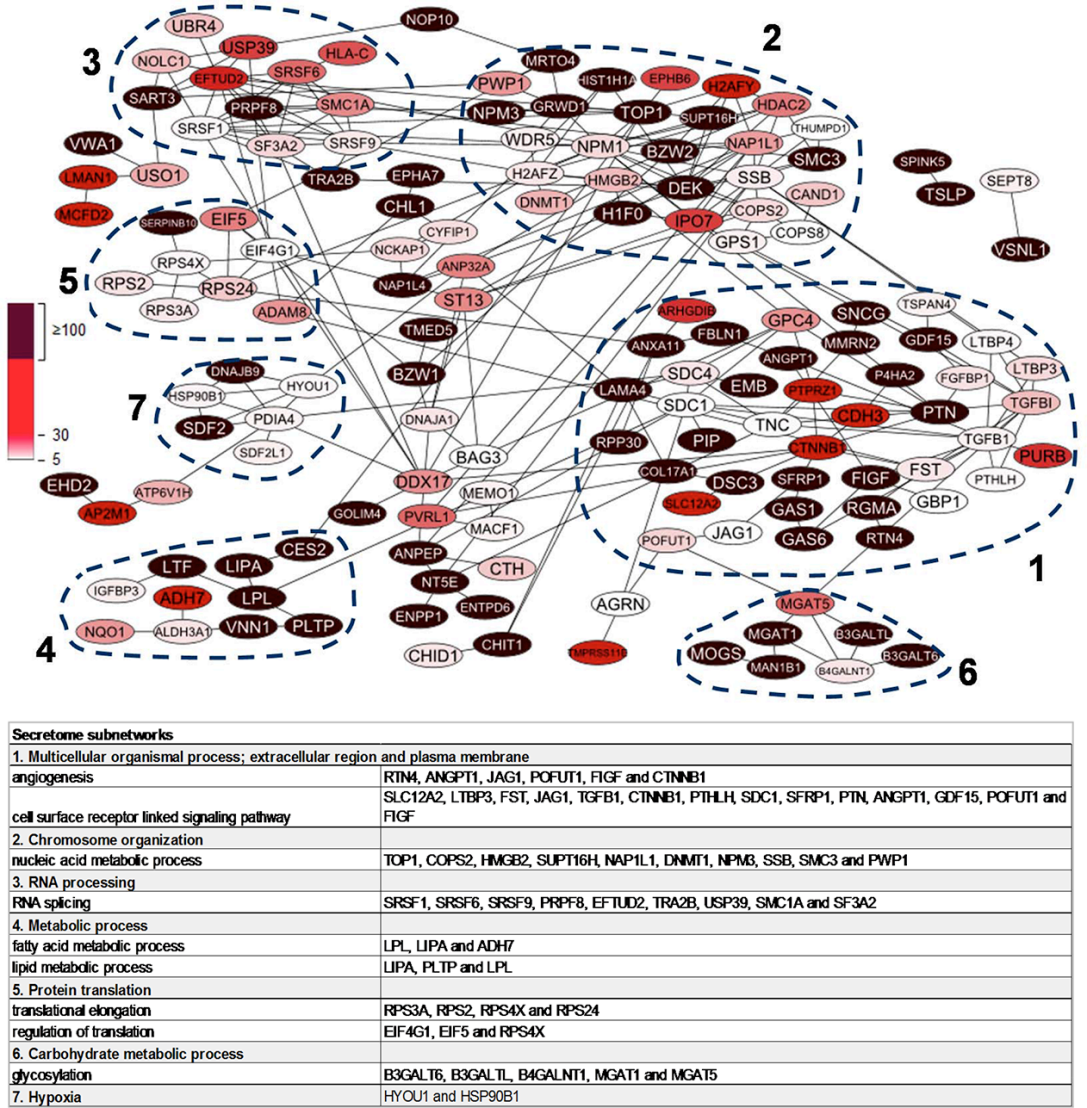
Protein-protein interaction network of 215 highly upregulated proteins in *BRCA1*-deficient relative to *BRCA1*-proficient secretomes Nodes represent proteins while the edges represent direct (physical) and indirect (functional) associations. Dashed lines indicate top seven most populated clusters identified by ClusterViz cluster analysis. Table shows representative biological processes of the seven clusters according to BinGO gene ontology analysis.

To explore the connectivity and biological functions of the 215 highly discriminatory *BRCA1*-deficient proteins, we used the STRING tool in conjunction with gene ontology (GO) analysis. Detailed GO results are shown in Supplementary Table 3. This analysis yielded one large network of 155 proteins that consisted of seven regions of densely connected proteins (nodes) using the ClusterViz tool (minimum size of five proteins, Figure 3). These sub-networks were associated with the following biological processes as determined by BinGO analysis: 1. “multi-cellular organismal process” with sub-terms “angiogenesis” and “cell surface receptor-linked signaling pathway”; 2.”chromosome organization” and “nucleic acid metabolic process” including the BRCA-like signature protein TOP1, a protein involved in DNA topological processes, and nucleophosmin (NPM1), a protein functionally associated with BRCA1-deficient double-stranded DNA repair; 3. “RNA processing” and “RNA splicing”, including PRPF8 and SMC1A, that are both part of the previously published 45 protein BRCA-like tissue signature; 4. “metabolic process” with sub-terms “fatty acid metabolic process” and “lipid metabolic process”; 5. a cluster centered around protein translation with five out of eight proteins involved in the biological process “translation” (more specifically in “translational elongation”) and three in “regulation of translation”; 6. “carbohydrate metabolic process”; 7. this cluster had no clear association with any biological processes, but two proteins, HYOU1 and HSP90B1, are involved in response to hypoxia (Figure 3).

The network of the 100 proteins highly abundantly released in *BRCA1*-proficient secretome is shown in Supplementary Figure 5 and contains three sub-networks associated with “metabolic process”, “cell adhesion” and “cellular component movement”.

In conclusion, the 215 proteins highly enriched in the *BRCA1*-deficient secretome are involved in cell-cell contact and communication, chromatin processes, RNA processing, protein translation and include several proteins that have been linked to *BRCA1*-deficient breast cancer. *BRCA1*-proficient cells release far less proteins and comprise a limited and totally different set of functions.

### Human plasma proteome

We integrated the 215 highly abundant proteins of the *BRCA1*-deficient secretome into the public database of the human plasma proteome. Among these proteins, 162 (75%) were identified in plasma (Supplementary Table 2). This *in silico* analysis indicated the potential of most proteins to enter the blood circulation and may assist in prioritizing candidate markers for future studies.

### Exosome-like EVs as carriers of *BRCA1*-deficient proteins

**Figure 4:**
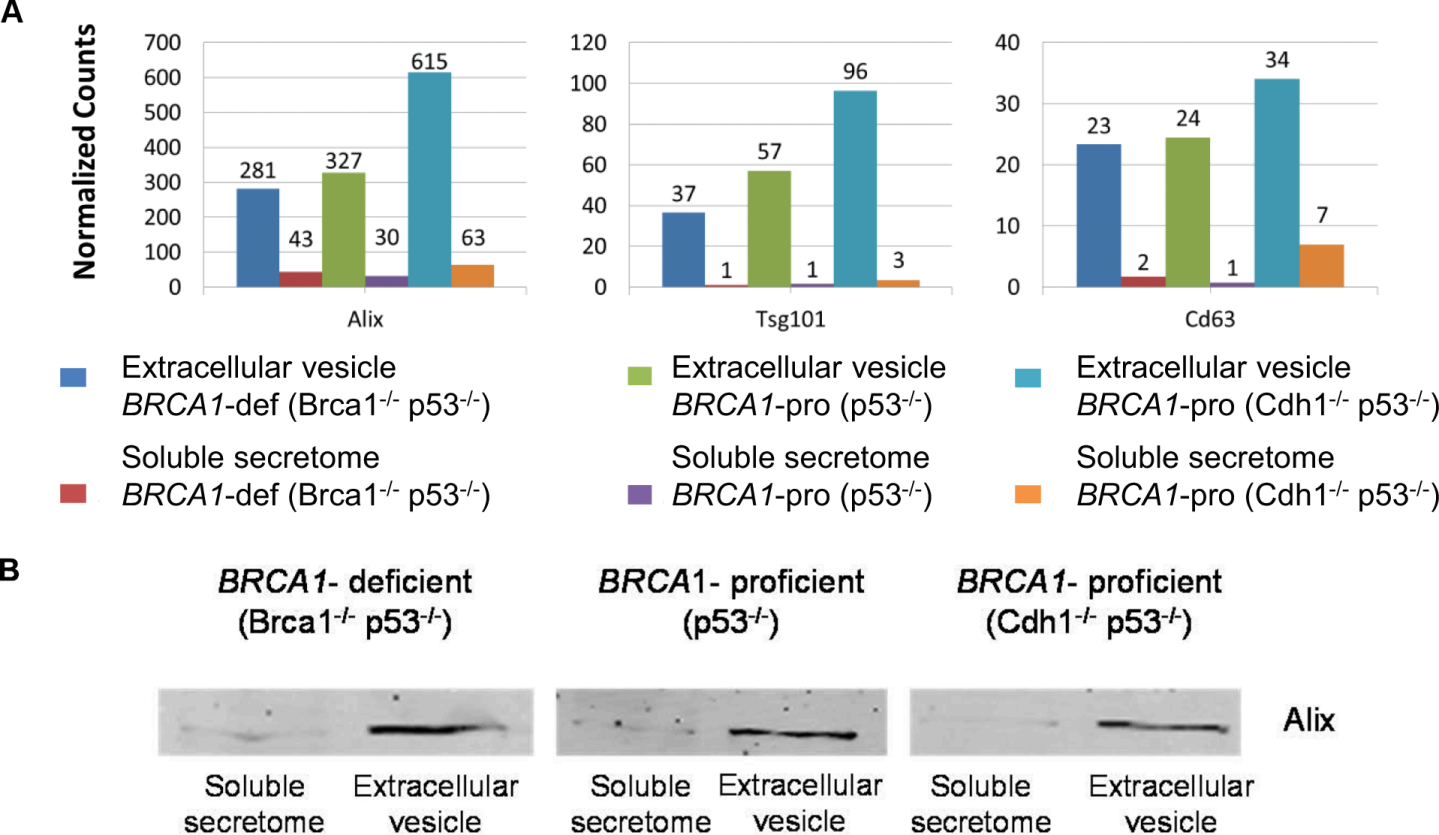
Exosome-associated proteins identified in soluble secretome and extracellular vesicle fractions (**A**) Quantification of exosome-associated proteins is presented by normalized spectral counts. (**B**) Western blot of Alix in soluble secretome and extracellular vesicle fractions. Cropped images of Western blot showing a common exosome marker (Alix) in soluble secretome and extracellular vesicle fractions.

As mentioned above, many proteins detected in the secretomes were predicted to be non-classically secreted. Among them, a large proportion was of nuclear origin. This observation prompted us to explore whether these proteins might be released via the non-classical, vesicle-mediated secretory pathway. Therefore, we performed proteomics of EVs isolated from the same *BRCA1*-deficient and *BRCA1*-proficient breast tumor cell lines resulting in 2,149 proteins (Supplementary Figure 6 and Supplementary Table 4). Exosomes are a class of EVs of endosomal origin. Multiple exosome-associated proteins were present and most proteins, including Alix, Tsg101 and CD63, were highly enriched in the EV fraction relative to the soluble secretome fraction (Figure 4A). Exosome enrichment was also confirmed by Western blot analysis of Alix (Figure 4B). The key biological processes (protein transport and vesicle-mediated transport) and molecular functions (GTP and ribonucleotide binding) were in line with the presence of several Rab GTases, known to be involved in vesicle formation, trafficking and transport [13] (Supplemental Figure 7).

A preliminary comparison of EV proteomes between *BRCA1*-deficient and -proficient groups identified 490 differentially abundant proteins. A total of 405 proteins were enriched in *BRCA1*-deficient vesicles. Major biological pathways of these 405 *BRCA1*-deficient proteins included DNA replication, RNA degradation and RNA splicing (Supplementary Figure 8).

A relatively large number of nuclear proteins was present among the 405 *BRCA1*-deficient proteins (Supplementary Figure 9). Overlap analysis revealed that 111 proteins were upregulated in the *BRCA1*-deficient secretome as well as in the *BRCA1*-deficient EVs relative to *BRCA1*-proficient counterparts. Among them were several nuclear proteins, which have previously been identified in *BRCA1*-deficient breast carcinomas (TOP1, SMC3, SSRP1 and DHX9, Supplementary Figure 10). Moreover, EV-mediated release of nuclear proteins was exemplified by TOP1, which was enriched in secretome and EVs of *BRCA1*-deficient group and its abundance was higher in EVs than in the secretome (Supplementary Figure 11). Taken together, intracellular (nuclear) proteins of *BRCA1*-deficient breast tumor cells (e.g. TOP1) released through exosome-like EVs may represent putative candidates related to *BRCA1*-deficient breast cancer.

### Validation of CHD3 and TOP1 in human breast carcinomas

Two proteins highly abundant in the *BRCA1*-deficient secretome, namely CDH3 and TOP1, were selected for validation in tumor tissue samples of breast cancer patients. CDH3, a plasma membrane protein, is involved in cell adhesion and has been proposed as a tissue marker in human *BRCA1*-deficient breast cancer as well as a serum marker in basal-like breast cancer [14, 15]. TOP1 is a nuclear protein involved in DNA replication and was selected for validation because of its high abundance in *BRCA1*-deficient secretome and EVs compared to *BRCA1*-proficient counterparts. Moreover, TOP1 is present in our previously identified BRCA-like tissue signature.

Protein expression of CDH3 and TOP1 was analysed in a panel of 253 human breast carcinomas comprising 102 *BRCA1-*related, 49 *BRCA2-*related and 102 sporadic breast carcinomas. Clinical and tumor characteristics are shown in Table 1. The majority of tissue samples concerned ductal carcinomas (86–90%). Significant differences among the three groups were noted with respect to age of histological diagnosis (*p* < 0.001), histological grade (*p* = 0.003), estrogen receptor (*p* < 0.001), progesterone receptor (*p* < 0.001) and HER2 expression (*p* = 0.039).

**Table 1:** Clinical and tumor characteristics of breast carcinoma tissues

	Sporadic breast cancer (*n* = 102) *n* (%)	*BRCA1*-mutated breast cancer (*n* = 102) *n* (%)	*BRCA2*-mutated breast cancer (*n* = 49) *n* (%)	*P* value
**Tumor type**				
Ductal	88 (86)	89 (87)	44 (90)	0.088
Lobular	10 (10)	4 (4)	3 (6)	
Medullary	0	4 (4)	0	
Other	4 (4)	5 (5)	2 (4)	
**Age**				
< 45	16 (16)	62 (62)	18 (37)	< 0.001
≥ 45	86 (84)	38 (38)	30 (63)	
**Grade**				
1	8 (8)	3 (3)	0	0.003
2	32 (34)	17 (18)	18 (39)	
3	55 (58)	77 (79)	28 (61)	
**Estrogen receptor**				
Positive	85 (83)	27 (27)	36 (75)	< 0.001
Negative	17 (17)	73 (73)	12 (25)	
**Progesterone receptor**				
Positive	61 (60)	17 (17)	22 (46)	< 0.001
Negative	41 (40)	84 (83)	26 (54)	
**HER2**				
Positive	11 (11)	2 (2)	3 (6)	0.039
Negative	91 (89)	98 (98)	45 (94)	
**TOP1**				
Positive	24 (35)	56 (60)	26 (59)	0.004
Negative	45 (65)	38 (40)	18 (41)	
**CDH3**				
Positive	17 (22)	68 (76)	14 (37)	< 0.001
Negative	62 (78)	22 (24)	24 (63)	
**TOP1 and CDH3**				
TOP1 and CDH3 positive	5 (7)	43 (48)	9 (24)	< 0.001
TOP1 and/or CDH3 negative	63 (93)	46 (52)	29 (76)	

Receptor status (ER, PgR and HER2) was completed in 250 samples. About 74% of *BRCA1*-related breast carcinomas, 23% of *BRCA2*-related breast carcinomas and 13% of sporadic breast carcinomas had triple negative phenotype lacking ER, PgR and HER2 expression (*p* < 0.001). A significantly higher rate was found in *BRCA1*-related compared with *BRCA2*-related breast carcinomas (*p* < 0.001) as well as with sporadic carcinomas (*p* < 0.001), while no difference was found between *BRCA2*-related breast carcinomas and sporadic breast carcinomas (*p* = 0.11). Triple negative breast cancer appeared to be diagnosed in women of younger age (< 45 years) compared with those aged ≥ 45 years (58% vs 27%, *p* < 0.001).

**Figure 5:**
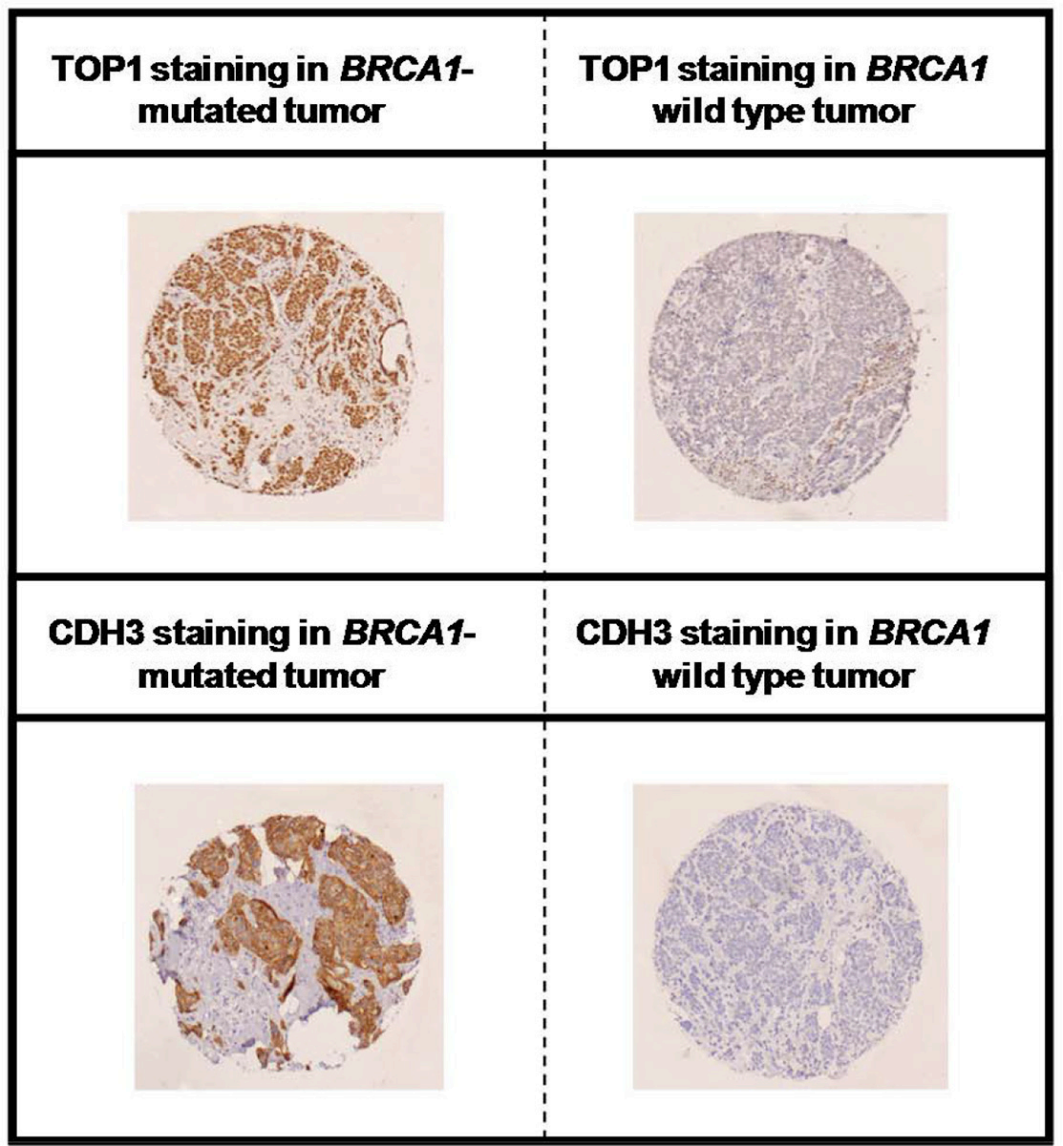
Immunohistochemical staining of TOP1 and CDH3 in *BRCA1*-deficient and proficient breast cancer Representative positive and negative staining of TOP1 (upper panel) and CDH3 (lower panel) are shown.

Representative staining results for TOP1 and CDH3 are shown in Figure 5 (high quality image of TOP1 and CDH3 staining is available in Supplementary Figure 12). The rates of TOP1-positive tumors were significantly different among the three groups (*p* = 0.004). TOP1 overexpression was mostly observed in *BRCA1*-related (60%) and in *BRCA2*-related breast carcinomas (59%) as compared to sporadic breast carcinomas (35%). Comparison between *BRCA1*-related and sporadic breast carcinomas demonstrated a significant difference in TOP1 expression (*p* = 0.002). With regard to CDH3, a significant difference was found among the three groups with 76% of *BRCA1*-related, 37% of *BRCA2*-related and 22% of sporadic breast carcinomas being CDH3 positive (*p* < 0.001). There was a significant difference between *BRCA1*-related and sporadic breast carcinomas in terms of CDH3-positive cases (*p* < 0.001). Of 195 breast cancer samples with TOP1 and CDH3 available, 57 samples were positive for TOP1 and CDH3 and 138 samples were TOP1 and/or CDH3 negative (79 positive for either of the two markers and 59 samples negative for both markers). When both markers were combined, significant differences in the rate of TOP1- and CHD3-positive tumors were found among the three groups (*p* < 0.001). Of note, the rates of TOP1 and CDH3 positivity were the highest in *BRCA1*-related breast carcinomas (48%) followed by *BRCA2*-related (24%) and sporadic breast carcinomas (7%).

Multiple logistic regression was performed to estimate the predictive effects of TOP1 or CDH3 for *BRCA1/2*-related breast carcinomas when adjusted for triple negative breast cancer and age (Table 2). We chose to use *BRCA1/2*-related breast carcinomas as an endpoint because both *BRCA1/2* mutations confer an increased risk of breast cancer. TOP1 expression was independently associated with *BRCA1/2*-related breast carcinomas (adjusted odds ratio [OR] 3.75; 95% confidence interval [95% CI], 1.82–7.71, *p* < 0.001). Similarly, an independent association of CDH3 expression for *BRCA1/2*-related breast carcinomas was found (adjusted OR 2.44; 95% CI, 1.08–5.49, *p* = 0.032). When TOP1 and CDH3 were combined, there was a significant association between breast carcinomas with positive TOP1 and CDH3 and *BRCA1/2* mutations (adjusted OR 5.05; 95% CI, 1.75–14.6, *p* = 0.003).

**Table 2:** Multiple logistic regression of TOP1 and CDH3 for BRCA1/2-related breast carcinomas

Independent variable	Beta	Standard error	Adjusted odds ratio (95% CI)	*P* value
Constant	−1.10	0.31		< 0.001
TNBC (yes vs no)	2.09	0.43	8.07 (3.46–18.8)	< 0.001
Age (< 45 vs ≥ 45 yrs)	1.59	0.41	4.88 (2.18–11.0)	< 0.001
TOP1 (positive vs negative)	1.32	0.37	3.75 (1.82–7.71)	< 0.001
Constant	−0.80	0.23		< 0.001
TNBC (yes vs no)	1.41	0.46	4.08 (1.64–10.1)	< 0.001
Age (< 45 vs ≥ 45 yrs)	1.46	0.39	4.30 (2.00–9.24)	< 0.001
CDH3 (positive vs negative)	0.89	0.41	2.44 (1.08–5.49)	0.032
Constant	−0.72	0.24		< 0.001
TNBC (yes vs no)	1.66	0.43	5.28 (2.28–12.2)	< 0.001
Age (< 45 vs ≥ 45 yrs)	1.46	0.41	4.31 (1.93–9.63)	0.003
TOP1 and CDH3 (positive vs TOP1 and/or CDH3 negative)	1.62	0.54	5.05 (1.75–14.6)	0.003

TNBC, triple-negative breast cancer.

## DISCUSSION

With proteomic analysis of breast tumor GEMM-derived secretomes, we identified a series of released proteins associated with *BRCA1* status. Most of these proteins are detectable in the blood circulation according to the human plasma proteome database, suggesting their clinical usefulness in blood-based testing for the prediction of *BRCA1* deficiency. Increased levels in TOP1 and CDH3 were validated in a large population-based series of breast cancer patients with a *BRCA1/2* mutation. These data merit further investigation.

The applicability of our results from murine breast tumor models to human *BRCA1*-related breast cancer is well supported by several levels of evidence. Many *BRCA1*-deficient candidate proteins identified play a role in DNA repair and are associated with *BRCA1* function. These included NPM1 [16], SMC1A [17], SMC3 [18], TOP1 [19], HMGB2 [20], DNMT1 [21], SRSF1 [22], EIF4G1 [23] and HDAC2 [24]. Further support was provided by our *in silico* analysis showing that *BRCA1* candidate proteins, when mapped to mRNA transcripts, could cluster *BRCA1*- and *BRCA2*-related breast cancer cases. More importantly, we validated two candidate proteins demonstrating, in accordance with animal data, increased expression in human *BRCA1*-related breast carcinomas. Finally, underscoring the potential use as non-invasive biomarker, 75 of protein candidates found in secretome including TOP1 and CDH3, are detectable in human plasma [25, 26]. Together, our data demonstrate the potential usefulness of breast tumor GEMM-derived secretome biomarkers for non-invasive diagnosis of human *BRCA1*-deficient breast cancer.

Interestingly, several nuclear-specific proteins were more abundantly present in the *BRCA1*-deficient compared to the *BRCA1*-proficient secretome. We demonstrated that exosome-like EVs may account for non-classical secretion of these nuclear proteins. EVs and especially exosomes have previously been implicated in many cellular functions, including cell-cell communication, tumor growth, metastases formation and angiogenesis [27, 28]. EVs have also been proposed to facilitate the intercellular transfer of a spectrum of cell-type specific factors e.g. miRNA and proteins. Interestingly, the molecular content of EVs has been shown to reflect the cells of origin, thereby underscoring the potential of EV protein profiling for the use of *BRCA1* detection.

In the present study, TOP1 was detected at an increased level in the *BRCA1*-deficient secretome and exosome-like vesicles as well as in human *BRCA1*-related breast carcinomas. Furthermore, multiple logistic regression analysis showed that TOP1 positivity was significantly associated with *BRCA1/2* mutations (adjusted OR = 3.75, *p* < 0.001). This association was independent of the presence of TNBC and age suggesting that assessment of TOP1 may substantially improve prediction of *BRCA1/2* mutations, in particular in breast carcinomas with hormone-receptor positive or HER2 positive breast cancer diagnosed in women aged ≥ 45 years. Our findings are in line with preclinical data reporting the involvement of TOP1 in DNA single-strand break repair in conjunction with BRCA1. In this regard, TOP1 has been described as a prominent target in *BRCA1*-deficient breast cancer [29]. TOP1 inhibitors indirectly induce DNA damage and are currently under investigation for their potential in targeting tumors with homologous recombination defects [30]. Moreover, TOP1 degradation has been described to be mediated by BRCA1 and accumulation of TOP1 has been observed in case of BRCA1 dysfunction [31]. Here, we identified TOP1 to be preferentially present in *BRCA1*-deficient EVs. Therefore, it may be speculated that accumulated TOP1 is partly due to release via EVs.

We demonstrated increased CDH3 levels in a large panel of human *BRCA1*-related breast carcinomas. Similar to TOP1, increased CHD3 expression was associated with *BRCA1/2*-related breast carcinoma, independent of TNBC and age. These result confirmed its potential as marker for *BRCA1/2* mutations. In previous studies, an increased level of CDH3 has been detected in a small cohort of *BRCA1*-deficient breast cancer [15], as well as in nipple aspirate fluid and serum obtained from women with basal-like breast tumors [14]. Gorski et al. [25] have shown that BRCA1 is a transcriptional repressor of CDH3, thereby providing a biological explanation for our observation of the strong presence of CDH3 in *BRCA1*-deficient breast carcinomas and large abundance in *BRCA1*-deficient secretome.

## MATERIALS AND METHODS

### Mouse models and cell line isolation and culture

*BRCA1*-deficient breast tumors from K14*Cre*;*Brca*^−/−^;*p53*^−/−^ GEMMs resemble human *BRCA1*-deficient breast cancer as determined by histopathology and genotyping. *BRCA1*-proficient breast tumors from K14*Cre*;*p53*^−/−^ GEMMs resemble human sporadic basal-like breast cancer, whereas *BRCA1*-proficient breast tumors from K14*Cre*;*Cdh1*^−/−^;*p53*^−/−^ GEMMs are histopathologically similar to human pleomorphic lobular breast cancer [10].

Primary breast tumor cells were isolated from tumor-bearing GEMMs as described in detail elsewhere [32]. Cell lines were cultured at 37°C and 5% CO_2_ atmosphere in DMEM/F12-GlutaMAX medium (Gibco) supplemented with 10% fetal calf serum (FCS), 1% penicillin-streptomycin (Lonza), 5 μg/ml insulin, 5 ng/ml epidermal growth factor (Life Technologies) and 5 ng/ml cholera toxin. Serum-free medium contains the above-mentioned supplements without FCS. All animal experiments were approved by the animal research committee of the Netherlands Cancer Institute.

### Preparation of breast tumor cell line secretomes

Cell line secretomes were prepared as described before [11]. In brief, tumor cells were grown until 70–80% confluency and incubated in serum-free medium overnight for 22–24 h. The conditioned medium (referred to as secretome) was removed from cell debris and concentrated to a final volume of ~50 μL using 3 kDa cutoff spin filter (Millipore). The samples supplemented with Sodium Dodecyl Sulfate (SDS) sample buffer containing 100 mM dithiotreitol were stored at −80°C until further analysis.

### Extracellular vesicles isolation

Isolation of EVs was performed by ultracentrifugation according to an established method [33, 34]. In short, ~5 mL concentrated secretome was subjected to differential centrifugation steps at 12,000 × *g* for 1 h and at 110,000 × *g* for 2 h using a Beckman SW40TI rotor. The supernatant, further referred to as the soluble protein fraction, was collected and the EV pellet was centrifuged at 110,000 × *g* for another 2 h and suspended in SDS sample buffer before storage at −80°C. The soluble protein fraction was concentrated and stored at −80°C supplemented with SDS sample buffer.

### Western blot

EV and soluble protein fractions were immunoblotted with anti-Alix primary antibody (3A9, Cell signaling), followed by secondary antibody conjugated with IRDye 680 (Rockland). Fluorescent signals were detected with an Odyssey infrared imaging system (LI-COR Biosciences).

### Proteomics by tandem mass spectrometry and database searching

Proteins from secretomes or EVs were fractionated by one-dimensional electrophoresis followed by trypsin in-gel protein digestion and nanocapillary liquid chromatography-tandem mass spectrometry (GeLC-MS/MS) [11]. Secretome tryptic digests were measured on an Ultimate 3000 nanoLC system (Dionex LC-Packings) on-line coupled to the LTQ-FT hybrid mass spectrometer (Thermo Fisher) using a top5 MS/MS method. EV tryptic digests were measured on a Q Exactive mass spectrometer (Thermo Fisher) coupled to a Ultimate 3000 nanoLC system (Dionex LC-Packings). The raw MS/MS data were processed as described before [8].

### Tissue microarray and immunohistochemistry

The study group comprised 253 cases of human breast carcinomas, among which were 102 *BRCA1*, 49 *BRCA2* germline mutation *-*related cases and 102 breast cancer cases not known to have a *BRCA1/2* mutation (further denoted as “sporadic”). Four μm thick sections were stained with hematoxylin and eosin for histopathology. Tumor type was assessed according to WHO and tumors were graded according to the Nottingham grading system. Tissue microarrays were constructed and stained for estrogen receptor (ER, 1:100, DAKO), progesterone receptor (PR, 1:100, DAKO), human epidermal growth factor receptor-2 (HER2, 1:100, Thermo Scientific [Neomarkers]), TOP1 (1:100, Abcam) and CDH3 (extracellular domain, 1:400, BD Biosciences, Pharmingen). Appropriate positive and negative controls were used throughout.

All specimens were scored by a single pathologist (PJvD), who was blinded to the origin of tumors. For ER and PR, the percentage of positive nuclei was scored. Samples with ≥ 10% immunopositive malignant cells were classified as ER or PR positive. For HER2 and CDH3, membranous staining was scored according to the DAKO scoring system as 0, 1+, 2+ and 3+, considering HER2 3+ and CDH3 1+, 2+ and 3+ cases as positive. For TOP1, the percentage of positive nuclei and the intensity of staining were scored. The product of the percentage of positive nuclei and the intensity of staining was calculated and cases above 170 were classified as positive. Since we used archival pathology material, which does not interfere with patient care and does not involve the physical involvement of the patient, no ethical approval was required according to institutional and Dutch regulations under an opt out system (reviewed by van Diest et al. [35]).

### Data analysis

For each protein, spectral counts were normalized for the total spectral counts in each sample. The normalized spectral counts were used for further comparative analyses. Differences in relative protein abundance, expressed as normalized spectral count, between *BRCA1*-deficient and -proficient GEMMs were analyzed using the beta binomial test [36–38]. *P* < 0.05 was considered statistically significant. Hierarchical unsupervised clustering was carried out on all identified proteins. Associations between immunohistochemistry (IHC) expression of proteins were tested by Pearson's Chi-square test. Multiple logistic regression was used to estimate the predictive effects of candidate biomarkers for identifying mutation carriers while adjusting for confounding factors. All statistical analyses were carried out with SPSS and R.

The clinical relevance of protein candidates was evaluated by analyzing the gene expression dataset containing mRNA expression of *BRCA1/2*-related and sporadic breast carcinomas as published by Jönnson et al. [12]. As clustering parameters for the gene expression dataset, we used a Spearman rank correlation in combination with the Ward's distance. mRNA levels of candidate proteins between *BRCA1/2*-deficient breast cancer and sporadic breast cancer were evaluated with Mann-Whitney *U* test using *P* < 0.1 as an arbitrary cut-off. To identify putative blood-based markers, protein candidates were evaluated in different human plasma proteome databases [39, 40].

Ingenuity pathway analysis software (Ingenuity Systems) and DAVID tool [41] were used to analyze biological functions and deregulated pathways in *BRCA1*-deficient breast cancer. SignalP and SecretomeP were used to predict proteins potentially undergoing classical and non-classical secretion [13, 42]. Cytoscape (version 2.7.2) [43] and STRING tool (version 9.0) [44] were used to analyze protein interactions as well as for network analysis. Cytoscape ClusterViz plugin was employed to identify clusters of biological networks [45].

## CONCLUSIONS AND OUTLOOK

Our study provides a promising set of secreted or excreted proteins that could find use in early non-invasive detection of tumors that arise in *BRCA1* mutation carriers and that might support management of treatment choice. Further validation of the detection of released biomarker proteins in body fluids, such as blood or nipple aspirate fluid, is a vital aspect to be resolved for future clinical applications. At present, this validation is hampered by limited availability of sensitive ELISA assays for the majority of candidate protein biomarkers. Therefore, we here applied immunohistochemistry for verification of increased expression of TOP1 and CDH3 in *BRCA1*-deficient breast carcinomas. New multiplex technologies, such as targeted mass spectrometry, may provide better opportunities for future large-scale validation in biofluids. Importantly, up to 75% of the *BRCA1* deficiency-associated secretome proteins are detectable in blood, thereby underscoring the value of our proteomic strategy. Future studies are warranted to assess the non-invasive diagnostic power of these proteins for early detection of breast tumors in cohorts of *BRCA1* mutation carriers.

## SUPPLEMENTARY MATERIALS FIGURES AND TABLES










