# Supplementary material for: Secretome proteomics reveals candidate non-invasive biomarkers of BRCA1 deficiency in breast cancer
Source: Oncotarget. 2016 Aug 23;7(39):63537–48. doi: 10.18632/oncotarget.11535 (PMC5325383; doi:10.18632/oncotarget.11535)
Supplement: Supplementary file 1 [file oncotarget-07-63537-s001.pdf]

# Secretome proteomics reveals candidate non-invasive biomarkers of *BRCA1* deficiency in breast cancer

## Supplementary Materials

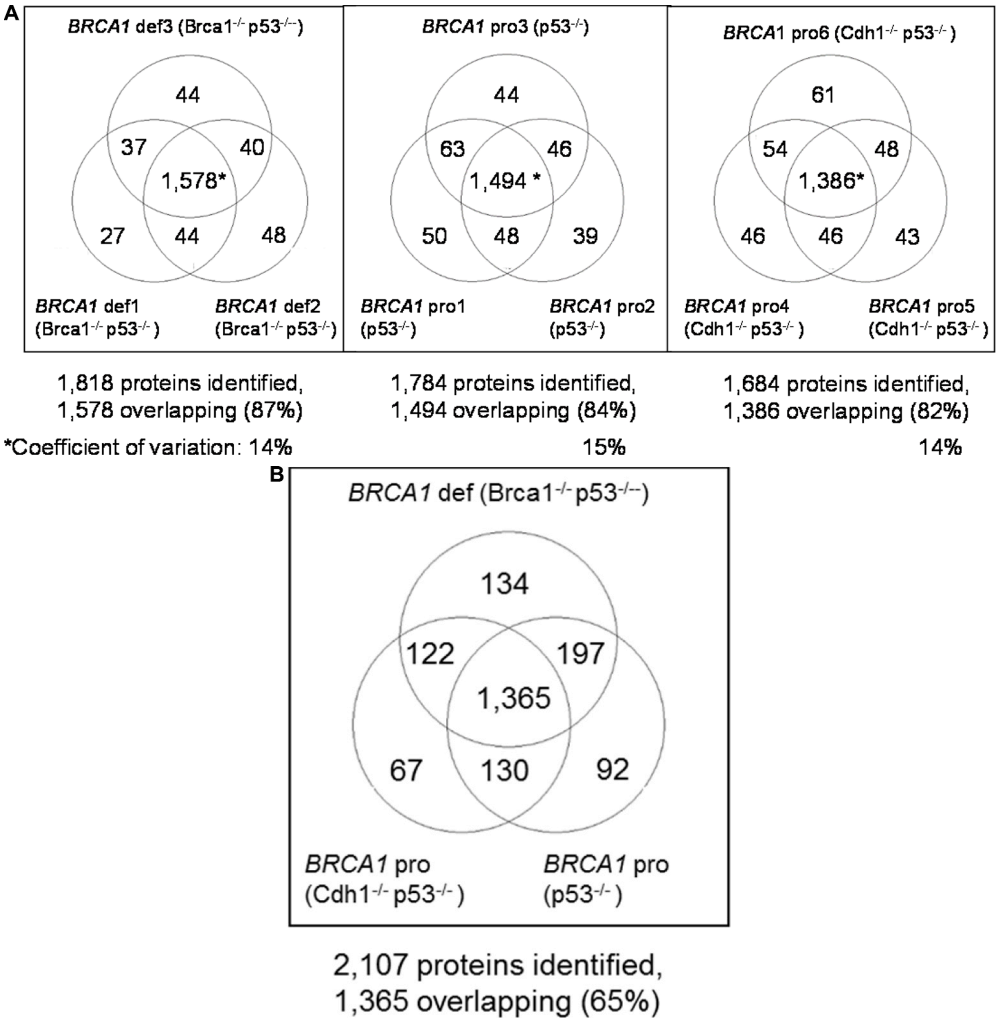


**Supplementary Figure S1:** (**A**) Venn diagram of protein identifications in three biological replicates. (**B**) Venn diagram of protein identifications among the three groups. Proteins identified in at least one triplicate sample of each group were used for overlap analysis.

***BRCA1* deficient (Brca1$^{-/-}$ p53$^{-/-}$ )**

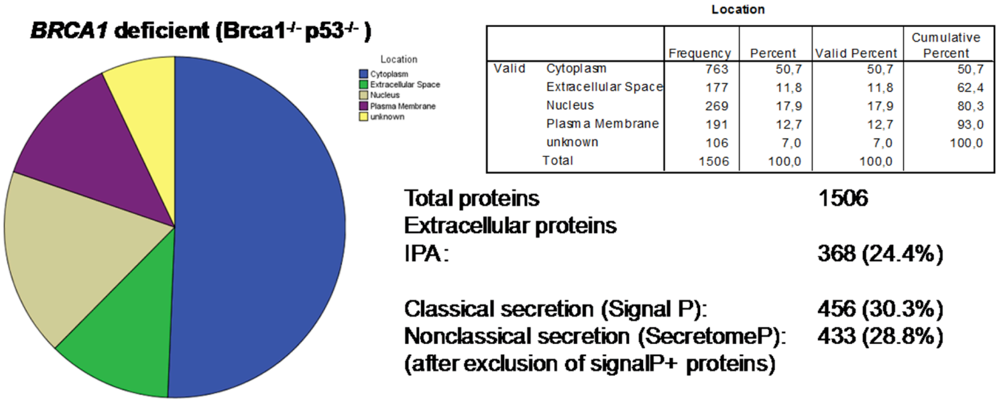


**Location**

| | | Frequency | Percent | Valid Percent | Cumulative Percent |
|---|---|---|---|---|---|
| Valid | Cytoplasm | 763 | 50,7 | 50,7 | 50,7 |
| | Extracellular Space | 177 | 11,8 | 11,8 | 62,4 |
| | Nucleus | 269 | 17,9 | 17,9 | 80,3 |
| | Plasma Membrane | 191 | 12,7 | 12,7 | 93,0 |
| | unknown | 106 | 7,0 | 7,0 | 100,0 |
| | Total | 1506 | 100,0 | 100,0 | |

Total proteins 1506
Extracellular proteins
IPA: 368 (24.4%)

Classical secretion (Signal P): 456 (30.3%)
Nonclassical secretion (SecretomeP): 433 (28.8%)
(after exclusion of signalP+ proteins)

***BRCA1* proficient (p53$^{-/-}$)**

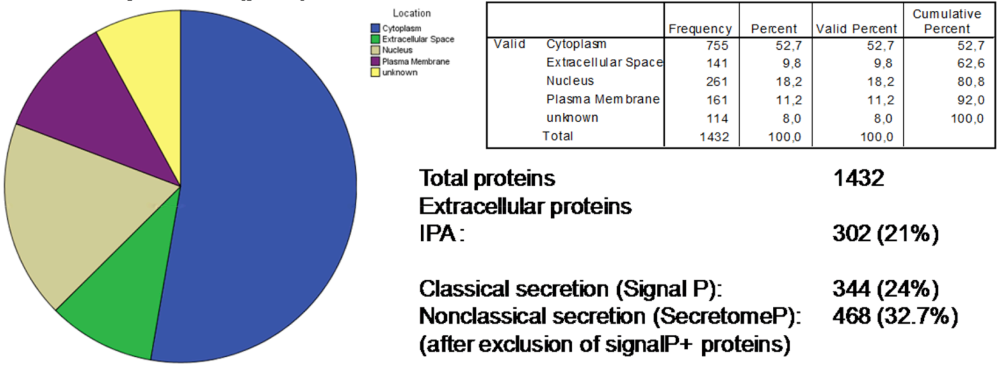


**Location**

| | | Frequency | Percent | Valid Percent | Cumulative Percent |
|---|---|---|---|---|---|
| Valid | Cytoplasm | 755 | 52,7 | 52,7 | 52,7 |
| | Extracellular Space | 141 | 9,8 | 9,8 | 62,6 |
| | Nucleus | 261 | 18,2 | 18,2 | 80,8 |
| | Plasma Membrane | 161 | 11,2 | 11,2 | 92,0 |
| | unknown | 114 | 8,0 | 8,0 | 100,0 |
| | Total | 1432 | 100,0 | 100,0 | |

Total proteins 1432
Extracellular proteins
IPA: 302 (21%)

Classical secretion (Signal P): 344 (24%)
Nonclassical secretion (SecretomeP): 468 (32.7%)
(after exclusion of signalP+ proteins)

***BRCA1* proficient (Cdh1$^{-/-}$ p53$^{-/-}$ )**

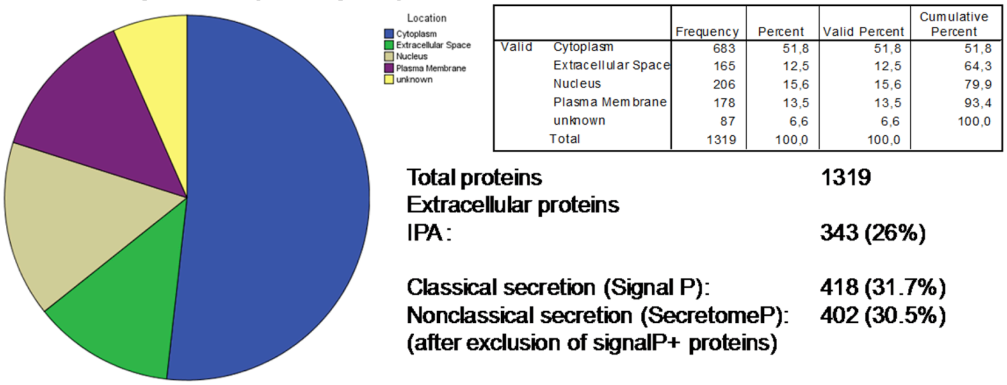


**Location**

| | | Frequency | Percent | Valid Percent | Cumulative Percent |
|---|---|---|---|---|---|
| Valid | Cytoplasm | 683 | 51,8 | 51,8 | 51,8 |
| | Extracellular Space | 165 | 12,5 | 12,5 | 64,3 |
| | Nucleus | 206 | 15,6 | 15,6 | 79,9 |
| | Plasma Membrane | 178 | 13,5 | 13,5 | 93,4 |
| | unknown | 87 | 6,6 | 6,6 | 100,0 |
| | Total | 1319 | 100,0 | 100,0 | |

Total proteins 1319
Extracellular proteins
IPA: 343 (26%)

Classical secretion (Signal P): 418 (31.7%)
Nonclassical secretion (SecretomeP): 402 (30.5%)
(after exclusion of signalP+ proteins)

**Supplementary Figure S2: Analysis of subcellular location, SignalP and SecretomeP**.

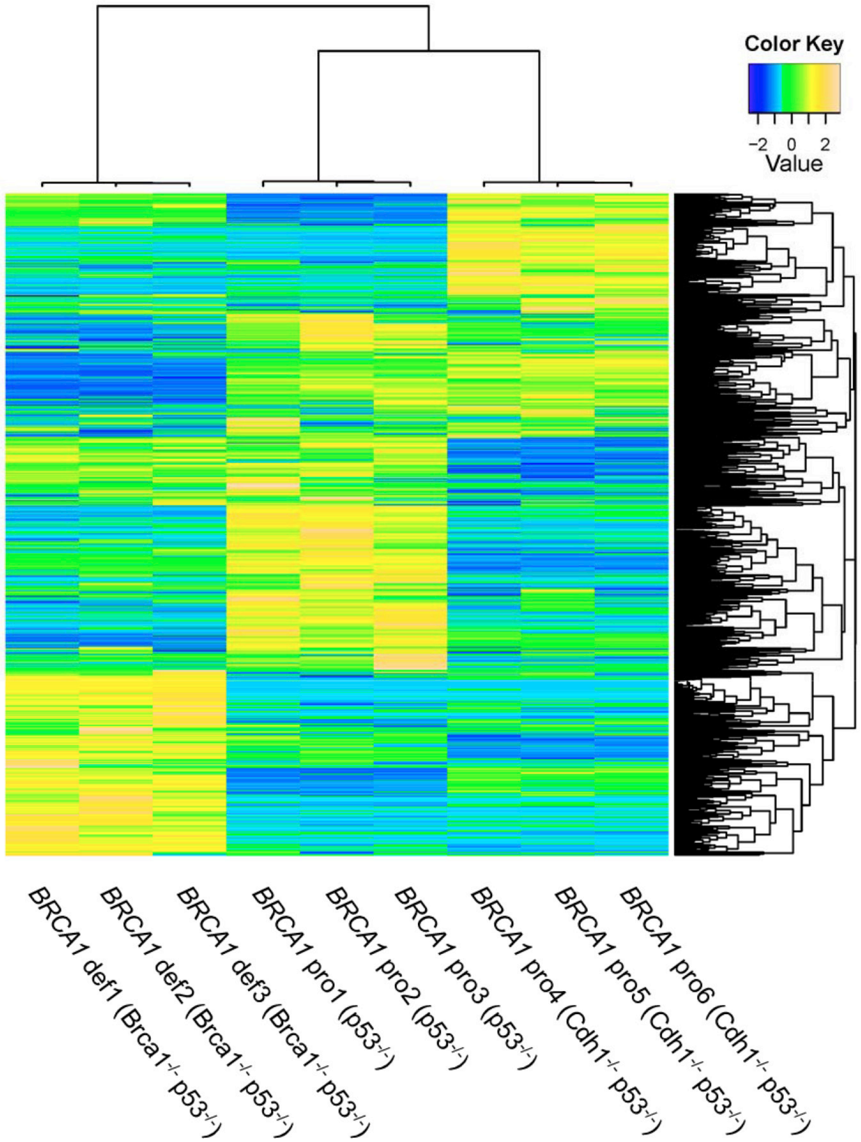


**Supplementary Figure S3: Unsupervised cluster analysis on all proteins identified in secretomes shows three distinct groups based on the mutational status**.

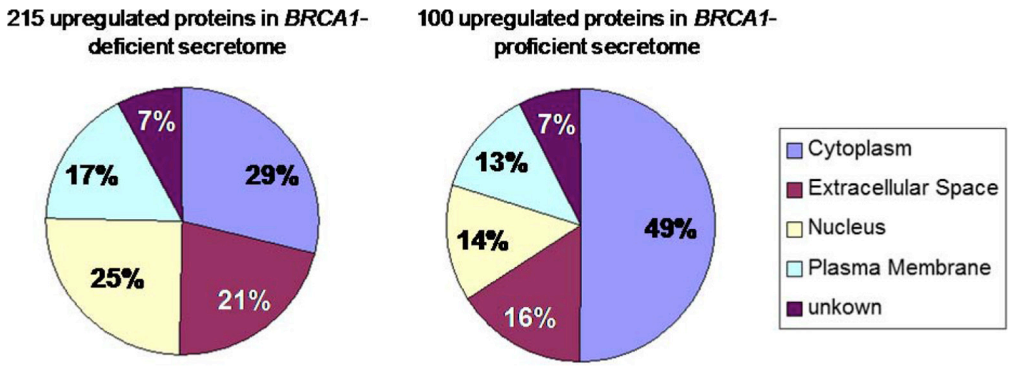


**Supplementary Figure S4: Gene Ontology analysis of subcellular localization of upregulated ($n$ = 215) and downregulated ($n$ = 100) proteins in BRCA1-deficient secretomes**.

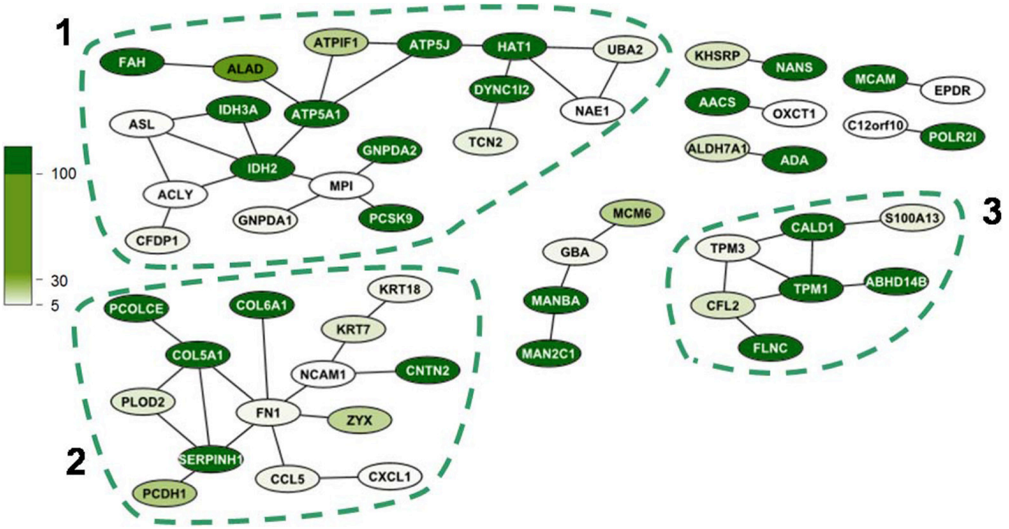


**Supplementary Figure S5: Biological networks of 100 highly downregulated proteins in BRCA1-deficient secretome**.

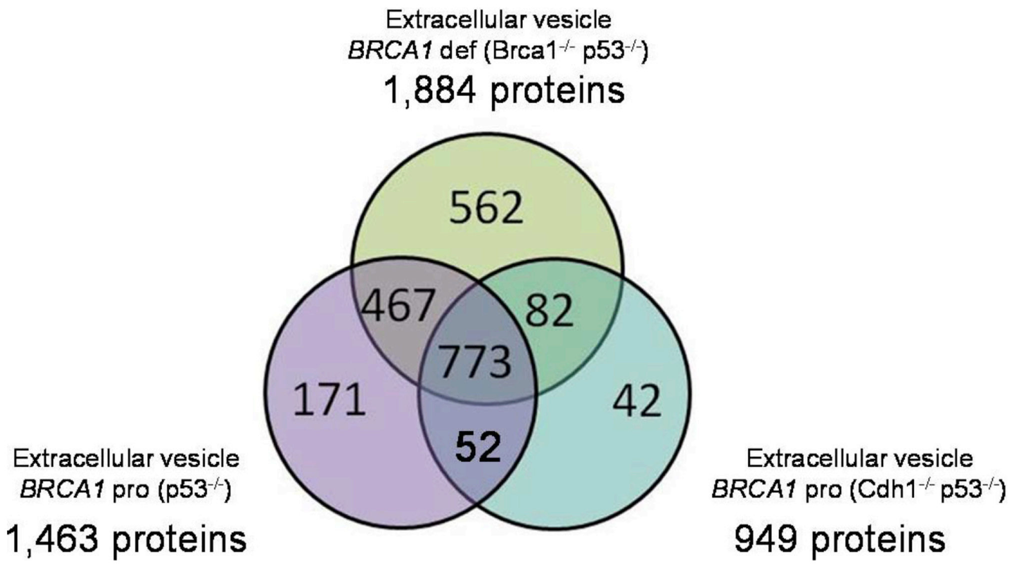


**Supplementary Figure S6: Venn diagram representing the total number of uniquely identified proteins in extracellular vesicles across three groups**.

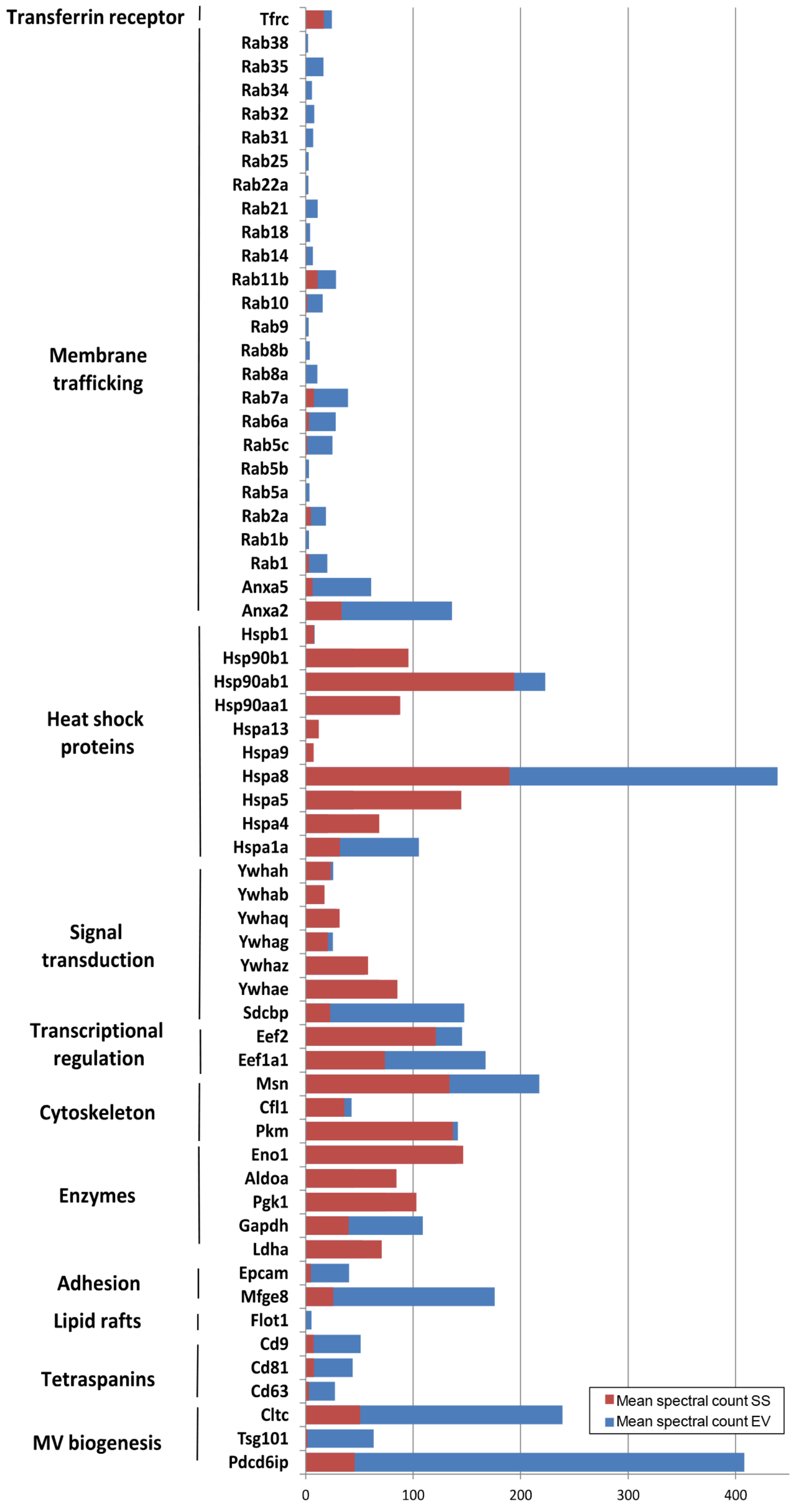


**Supplementary Figure S7: Distribution of microvesicle-associated proteins in extracellular vesicle (EV) and soluble secretome (SS) fractions identified across three experiments.**

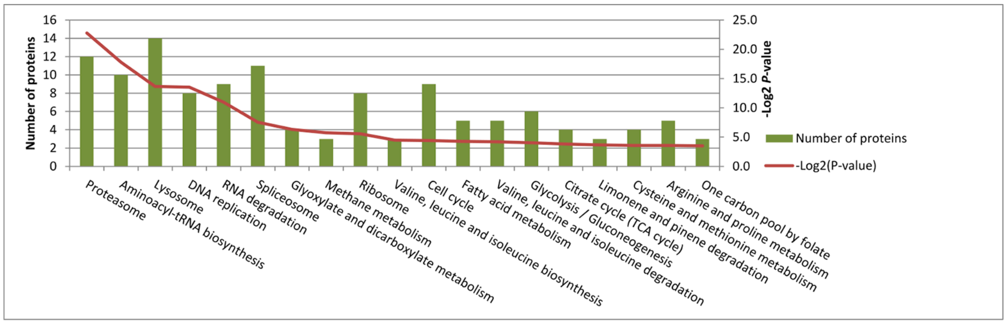


**Supplementary Figure S8: Significant biological processes in 405 upregulated proteins in extracellular vesicles of *BRCA1*-deficient breast tumor cell lines .**

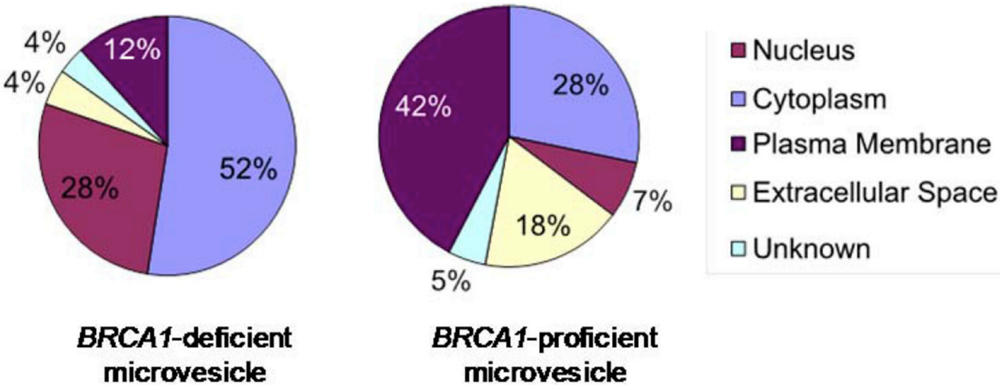


**Supplementary Figure S9: Subcellular localization of proteins enriched in *BRCA1*-deficient extracellular vesicles (*n* = 405, left panel) and in BRCA1-proficient extracellular vesicles (*n* = 85, right panel)**.

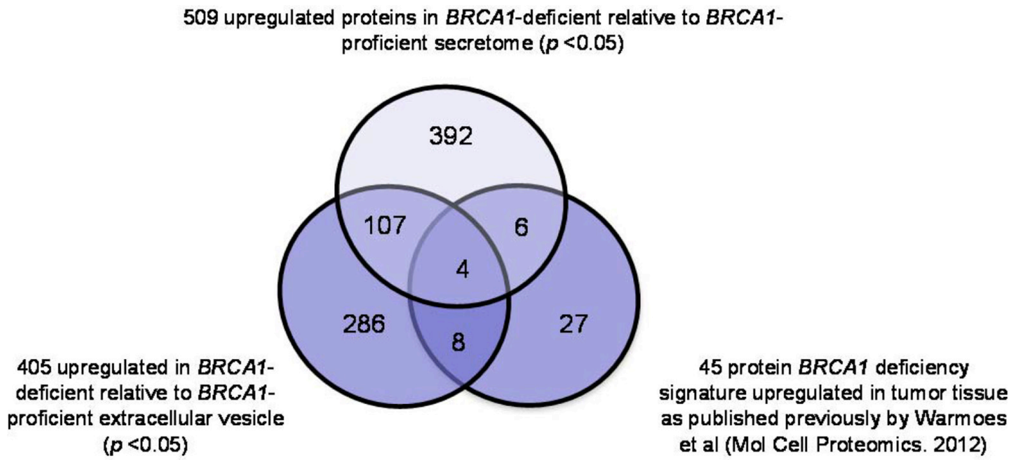


**Supplementary Figure S10: Overlap analysis of proteins enriched in *BRCA1*-deficient secretome, extracellular vesicle and *BRCA1*-deficient tissue signature**.

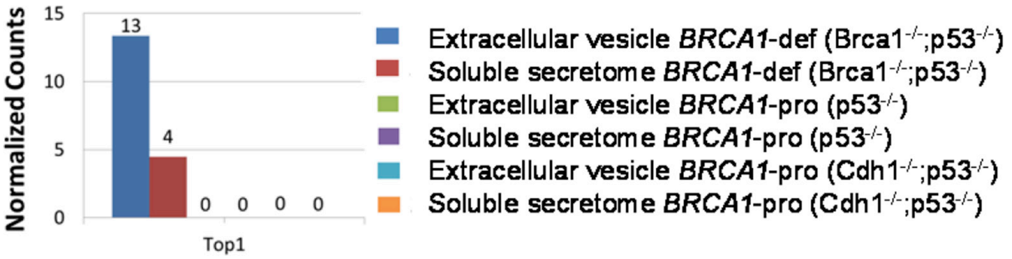


**Supplementary Figure S11: Levels of TOP1 in soluble secretome and microvesicle factions of individual samples**.

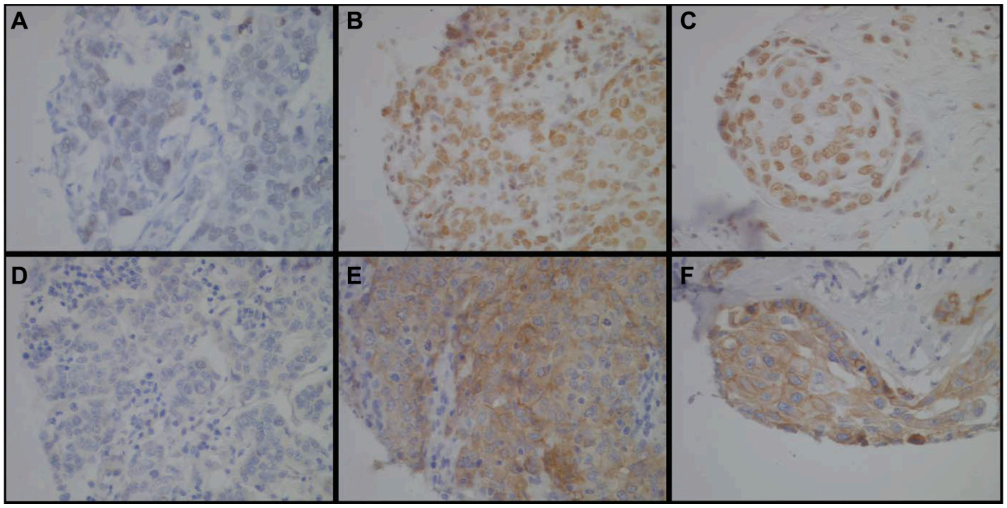


**Supplementary Figure S12: Representative TOP1 and CDH3 staining.** Representative TOP1 IHC positive staining of a sporadic (**A**) *BRCA1*- (**B**) and *BRCA2*-mutated (**C**) human breast cancer case and representative CDH3 positive staining of a sporadic (**D**), *BRCA1*-(**E**) and *BRCA2*-mutated (**F**) human breast cancer case.

**Supplementary Table S1: List of proteins detected in BRCA1-deficient and -proficient secretomes harvested from breast tumor cell lines and spectral count quantification data.** See Supplementary_Table_S1

**Supplementary Table S2: 509 upregulated proteins in BRCA1-deficient relative to BRCA1-proficient secretomes.** See Supplementary_Table_S2

**Supplementary Table S3: BinGO gene ontology of biological process analysis on sub-networks.** See Supplementary_ Table_S3

**Supplementary Table S4: List of proteins detected in BRCA1-deficient and -proficient extracellular vesicles and soluble secretome fractions and spectral count quantification data.** See Supplementary_Table_S4
